# Generation and characterization of a highly effective protein substrate for analysis of FLT3 activity

**DOI:** 10.1186/1756-8722-5-39

**Published:** 2012-07-16

**Authors:** Yun Chen, Yao Guo, Jiayu Han, Wanting Tina Ho, Shibo Li, Xueqi Fu, Zhizhuang Joe Zhao

**Affiliations:** 1 Department of Pathology, University of Oklahoma Health Sciences Center, Oklahoma City, OK, 73104, USA; 2 Department of Pediatrics, University of Oklahoma Health Sciences Center, Oklahoma City, OK, 73104, USA; 3 Edmond H. Fischer Signal Transduction Laboratory, College of Life Sciences, Jilin University, Changchun, China

**Keywords:** Tyrosine kinase, FLT3, Activity assay, Inhibitor screening, Acute myeloid leukemia

## Abstract

**Background:**

Gain-of-function mutations of tyrosine kinase FLT3 are frequently found in acute myeloid leukemia (AML). This has made FLT3 an important marker for disease diagnosis and a highly attractive target for therapeutic drug development. This study is intended to generate a sensitive substrate for assays of the FLT3 enzymatic activity.

**Methods:**

We expressed in *Escherichia coli* cells a glutathione S-transferase (GST) fusion protein designated GST-FLT3S, which contains a peptide sequence derived from an autophosphorylation site of FLT3. The protein was used to analyze tyrosine kinase activity of baculovirus-expressed FLT3 and crude cell extracts of bone marrow cells from AML patients. It was also employed to perform FLT3 kinase assays for FLT3 inhibitor screening.

**Results:**

GST-FLT3S in solution or on beads was strongly phosphorylated by recombinant proteins carrying the catalytic domain of wild type FLT3 and FLT3D835 mutants, with the latter exhibiting much higher activity and efficiency. GST-FLT3S was also able to detect elevated tyrosine kinase activity in bone marrow cell extracts from AML patients. A small-scale inhibitor screening led to identification of several potent inhibitors of wild type and mutant forms of FLT3.

**Conclusions:**

GST-FLT3S is a sensitive protein substrate for FLT3 assays. It may find applications in diagnosis of diseases related to abnormal FLT3 activity and in inhibitor screening for drug development.

## Background

FLT3 is a member of the class III receptor tyrosine kinase (RTK) family. It is expressed in immature hematopoietic cells and plays an important role in the normal development of stem cells and the immune system [1,2]. Mutations of FLT3 have been detected in approximately 30% of patients with acute myelogenous leukemia (AML) and in a small number of patients with acute lymphocytic leukemia or myelodysplastic syndrome [3,4]. The most common mutations of FLT3 found in hematopoietic malignancies involve internal tandem duplications (FLT3-ITD) within the juxtamembrane domain, while point mutations within the tyrosine kinase domain (FLT3-TKD) is found in about 7% of AML patients. Both types of mutations cause constitutive activation of FLT3 kinase activity, thereby turning on downstream signaling proteins and resulting in uncontrolled cell proliferation [3-5].

FLT3 mutations are important markers for AML diagnosis, and FLT3-ITD is strongly associated with the poor prognosis of AML patients [6]. In fact, detection of FLT3-ITD is currently a routine diagnostic practice, and the presence of FLT3-ITD guides therapeutic decisions in AML patients with a normal karyotype [7,8]. As gain-of-function mutants, FLT3-ITD and FLT3-TKD are obvious targets for therapeutic kinase inhibitors. Indeed, inhibiting FLT3 tyrosine kinase activity has been the focus of both preclinical and clinical research in AML. Many potent FLT3 inhibitors have been identified, and some have been clinically tested as single agents and in combination with chemotherapy, but thus far clinical responses have been limited [9,10]. Currently, sorafenib, an inhibitor of tyrosine protein kinases (VEGFR and PDGFR) and Raf kinases, is the only approved FLT3 inhibitor for clinical use. Sorafenib is available for off-label use although it does not usually lead to a complete response [11]. Further effort in FLT3 inhibitor screening is clearly needed. For this purpose, more effective methods for FLT3 kinase activity assays are highly desirable.

In this study, we have generated a glutathione S-transferase (GST) fusion protein carrying a peptide sequence derived from an autophosphorylation site of human FLT3. This protein designated GST-FLT3S, can be effectively phosphorylated by recombinant FLT3 enzymes. It can be used to detect elevated FLT3 activity in bone marrow cells from AML patients and to test inhibitory effects of various protein kinase inhibitors. We believe GST-FLT3S should find broad applications in detecting increased FLT3 activity from clinical samples for diagnostic purposes and for identifying effective FLT3 inhibitors through small-scale testing and large-scale screening.

## Results and discussion

### GST-FLT3S is a highly effective substrate for assays of FLT3 activity

**Figure 1:**
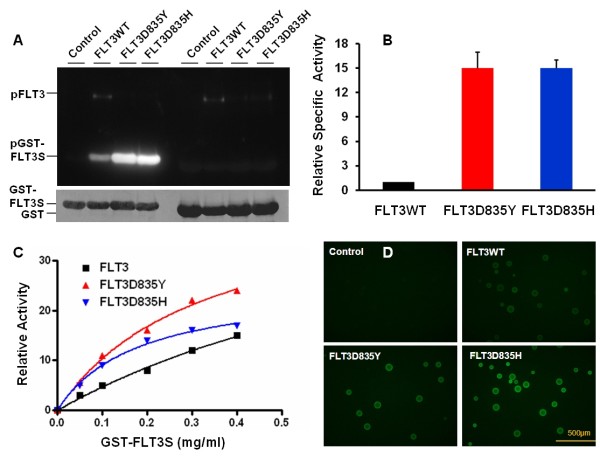
**GST-FLT3S is an effective substrate for FLT3 kinase activity assays.** Reactions were carried out with FLT3WT, FLT3D835Y, and FLT3D835H at 1.6, 0.4, and 0.4 μg/ml, respectively. **A.** Assays performed in the presence of 0.2 mg/ml GST-FLT3S or GST. Tyrosine phosphorylation was detected by using anti-phosphotyrosine antibody. Note that autophosphorylation of FLT3 was also seen. The protein levels of GST-FLT3S and GST were revealed by Coomassie blue staining. **B.** Comparison of specific activity of wild type and two mutant forms of FLT3 recombinant proteins determined with GST-FLT3S at 0.2 mg/ml. Error bars denote standard deviation. **C.** Activity assays performed with different concentrations of GST-FLT3S. **D.** Activity assays performed with GST-FLT3S immobilized on glutathione-Sepharose beads. Fluorescent images were acquired under fluorescent microscope with identical exposure times.

We constructed a pGex-GST-FLT3S plasmid, which encodes a GST fusion protein carrying the peptide sequence derived from the autophosphorylation site 589 of FLT3. When induced by 1 mM isopropy1 β-D-1-thiogalactopyranoside, *E. coli* cells transformed by the plasmid gave rise to a robust expression of GST-FLT3S in the exclusion body. From 1 liter of cell culture, over 50 mg of nearly homogeneous recombinant protein could usually be obtained by using a single glutathione-Sepharose column. For FLT3 kinase activity assays, we first expressed the catalytic domain of wild type and mutant forms of FLT3 as 6xHis-tagged recombinant proteins by using the baculovirus expression system. The recombinant proteins were purified from extracts of infected Sf9 cells through Ni-NTA-agarose columns. Figure 1 illustrates the results of FLT3 kinase activity assays. GST-FLT3S was strongly phosphorylated by recombinant proteins containing the catalytic domain of wild type and D835H and D835Y mutant forms of FLT3, while plain GST was not phosphorylated at all although it has 14 tyrosyl residues, demonstrating the specificity of the FLT3 kinase and phosphorylation of the FLT3 peptide fused to GST (Figure 1A). It should be noted that the mutant forms displayed much stronger phosphorylation of GST-FLT3S than wild type FLT3, although a lower amount of mutant enzymes were used in the assays. When normalized to protein expression level, FLT3D835Y and FLT3D835H exhibited 15-fold higher specific activity (Figure 1B). We further carried out reactions with different concentrations of substrates. The phosphorylation of GST-FLT3S obeys Michaelis–Menten kinetics with Km values of 1.1, 0.32, and 0.18 mg/ml GST-FLT3S for FLT3, FLT3D835H and FLT3D835Y, respectively (Figure 1C). The data indicates that the D835 mutants of FLT3 not only increase the catalytic turnover but also use the substrate more efficiently at lower concentrations. We further carried out the kinase assays with GST-FLT3S immobilized on glutathione-Sepharose beads and detected tyrosine phosphorylation using a fluorescein-labeled antibody. The data demonstrated consistent measurements of wild type and mutant FLT3 kinase activity (Figure 1D). This also provides a proof-of-principle for high throughput multiplex assays with multiple substrates immobilized on beads.

### GST-FLT3S can be used to detect increased tyrosine kinase activity in AML samples

**Figure 2:**
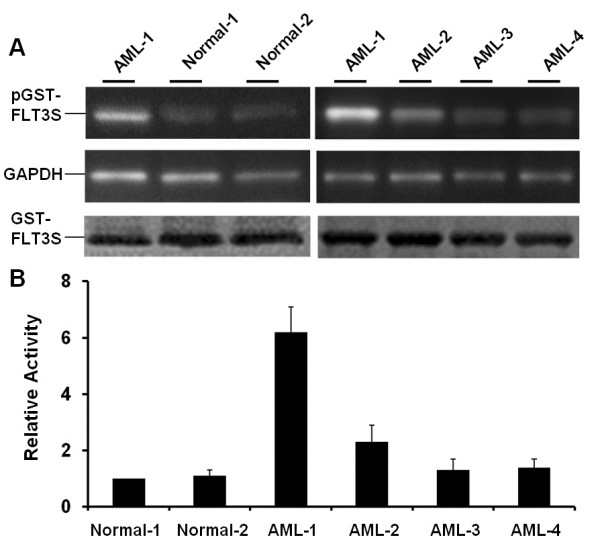
**Bone marrow samples from AML patients possess increased GST-FLT3S phosphorylation activities. A.** White blood cells from two normal and four AML bone marrow samples were extracted. Kinase assays were performed with cell extracts at 0.2 mg/ml in the presence of 0.2 mg/ml GST-FLT3S. Phosphorylation of GST-FLT3S was detected by using anti-phosphotyrosine, and equal protein loadings were revealed by anti-GAPDH (for house-keeping protein glyceraldehyde 3-phosphate dehydrogenase) and Coomassie blue staining (for GST-FLT3S). **B.** Quantitative representation of GST-FLT3S phosphorylation activity. Error bars denote standard deviation (n ≥ 3).

We employed GST-FLT3S to analyze cell extracts from 4 AML and 2 normal bone marrow samples. The assays identified 2 AML samples (AML1 and 2) with significantly increased phosphorylation of GST-FLT3S (p < 0.001, Figure 2). Interestingly, none of the four AML samples were found positive for the known FLT3-ITD and FLT3-D835 mutations. The elevated GST-FLT3S phosphorylation activity is likely caused by activation of FLT3 through other unknown mutations or mechanisms. Of course, we cannot rule out the involvement of other activated kinases which may also phosphorylate GST-FLT3S. Sample AML-1, which displayed over 6-fold increase in GST-FLT3S kinase activity, is cytogenetically normal as found with AML3 and 4. Sample AML-2 with over 2-fold increase in GST-FLT3S kinase activity was cytogenetically abnormal. GST-FLT3S thus serves as a unique tool for analyses of abnormal FLT3 and related kinase activities in patient samples. The assay appeared to be highly sensitive because a cell extract with 4 μg of total proteins is sufficient for each analysis. We believe that GST-FLT3S may be used for diagnoses of AML and other diseases involving elevated FLT3 activity. Further studies with more patient samples are warranted.

FLT3 mutations have been found in many patients with AML and ALL. Currently, various gene-based analyses have been developed to detect such mutations, and this has provided valuable information for diagnosis of the diseases and has helped in designing proper treatments [5-7]. However, the gene-based assays reveal only the presence, not the activity, of the mutant FLT3. Because GST-FLT3S directly detects enzymatic activity, it should identify abnormal elevations of the FLT3 activity due to mutations of the enzyme at unknown sites or to malfunction of other signaling components that regulate FLT3.

### GST-FLT3S can be used to screen FLT3 inhibitors

**Figure 3:**
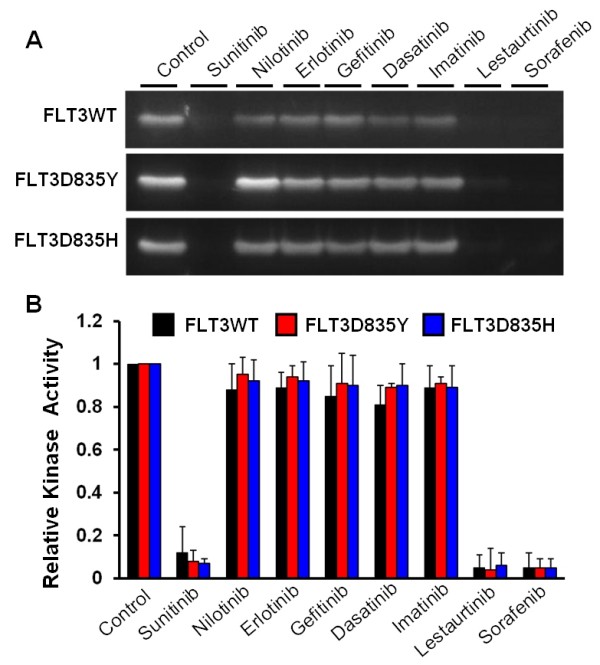
**Kinase assays with GST-FLT3S identify potent FLT3 inhibitors. A.** Tyrosine kinase activity of wild type and mutation forms of FLT3 were analyzed with 0.2 mg/ml GST-FLT3S in the presence of indicated tyrosine kinase inhibitors at 0.3 μM. Phosphorylation of GST-FLT3S was detected by using anti-phosphotyrosine antibody. **B.** Quantitative representation of GST-FLT3S phosphorylation activity. Error bars denote standard deviation (n ≥ 3). The stock solutions of inhibitors were made in dimethyl sulfoxide (DMSO), which was used as control. Note the strong inhibitory effects of sunitinib, sorafenib, and lestaurtinib.

Protein kinase inhibitors have found major applications in cancer therapies. With GST-FLT3S as a substrate, we analyzed the inhibitory effects of several known protein kinase inhibitors on FLT3 and its mutants. These inhibitors included sunitinib, nilotinib, erlotinib, gefitinib, dasatinib, imatinib, sorafenib, and lestaurtinib. Known targets of these inhibitors are listed in Table 1. Except for lestaurtinib, all are FDA-approved anticancer drugs targeting various tyrosine kinases. Data shown in Figure 3 demonstrated that sunitinib, sorafenib, and lestaurtinib caused over 90% inhibition of FLT3 activity at 0.3 μM while the rest had essentially no effect. These results were expected since these FLT3-inhibitory compounds are known to target multiple proteins kinases and have shown some efficacy in clinical trials of AML with FLT3-ITD [12,13]. Our study also demonstrated that these inhibitors target FLT3D835H and FLT3D835Y equally well. Therefore, GST-FLT3S could serve as a substrate for screening potential FLT3 inhibitors.

**Table 1:** Known targets of protein kinase inhibitors tested

**Inhibitors**	**Known Targets**
Sunitinib	VEGFRs, PDGFRs, KIT, and FLT3
Nilotinib	BCR-ABL, KIT, DDRs, PDGFRs, and CSF-1R
Erlotinib	EGFR and JAK2
Gefitinib	EGFR, Her2, and Her3
Dasatinib	BCR-ABL, SRCs, and KIT
Imatinib	BCR-ABL, KIT, and PDGFRs
Lestaurtinib	FLT3, JAK2, TrkA, TrkB, and TrkC
Sorafenib	B-Raf, C-Raf, PDGFRs, VEGFRs, KIT, and FLT3

Identifying potent inhibitors of oncogenic protein kinases is a new trend in anticancer drug development [9-11]. Our current study provides a unique protein substrate for FLT3 kinase assays to test inhibitory effects of existing protein kinase inhibitors and to screen chemical libraries for new inhibitors. It is suitable for assays with the substrate in solution or immobilized on a solid surface, in small scale or in high throughput. It allows direct measurement of phosphorylation of added proteins substrate with high sensitivity, serving as an alternative to inhibitor screening assays by determining phosphorylation of synthetic peptide substrates, autophopshorylation of kinases, and competition with ATP or analogs for binding to kinases.

## Conclusions

We have developed a robust protein substrate designated GST-FLT3S for assays of FLT3 kinase activity. GST-FLT3S can be produced economically in large quantities and used for assays of FLT3 in solution and when immobilized on beads. It reveals activation of FLT3 by mutations in the catalytic domain and detects increased tyrosine kinase activities in the cell extracts from AML patients. It can be used to test the effectiveness of existing protein kinase inhibitors of FLT3 and for large-scale inhibitor screening. We believe GST-FLT3S should serve as an important tool for future studies related to FLT3 and for disease diagnosis and therapeutic drug development. Finally, our study also demonstrates that GST is an excellent carrier for expression of short peptide sequences to serve as substrates of protein kinases.

## Methods

### Materials and clinical samples

Monoclonal anti-phosphotyrosine antibody PY20 was purchased from BD Biosciences. Protein kinase inhibitors were from ChemieTek. De-identified normal and AML bone marrow samples were collected from local clinical laboratories. The samples were residues from routine cytogenetic tests. Institutional review board approval was obtained before these samples were collected and analyzed. The bone marrow samples were treated with red cell lysis buffer, and proteins were extracted from white cells with a whole-cell extraction buffer containing 25 mM β-glycerophosphate (pH 7.3), 5 mM EDTA, 2 mM EGTA, 5 mM β-mercaptoethanol, 1% Triton X-100, 0.1 M NaCl, 1 mM sodium vanadate, and a protease inhibitor cocktail (Roche Applied Science). Cell lysates were cleared by centrifugation in a microfuge at 13,000g, and clear extracts were used directly for FLT3 activity assays. The remaining pellets were used for DNA isolation by using phenol/chloroform extraction after digestion of proteins with proteinase K.

### Expression and purification of GST-FLT3S and recombinant FLT3

To make the GST-FLT3S construct, DNA oligos encoding a peptide with the sequence SDNEYFYVD were synthesized and ligated into the pGex-2T vector, following a similar strategy previously described for a JAK2 substrate [14]. The peptide sequence was derived from human FLT3 and contains the tyrosine 589 autophosphorylation site. The recombinant fusion protein was expressed in *E. coli* cells and then purified by using a glutathione-Sepharose column. We employed the baculovirus expression system to express various wild type and mutant forms of FLT3 as described for other tyrosine kinases [15]. In brief, a DNA fragment encoding the entire intracellular portion (amino acid residues 573–993) of human FLT3 was cloned into the pBluebacHis2 vector (Invitrogen). D835H and D835Y and FLT3 kinase domain mutations were introduced by using site-specific mutagenesis. The resultant plasmid DNAs, together with Bac-N-blue DNA, were used to transfect Sf9 insect cells to generate recombinant viruses according to manufacturer’s protocol (Invitrogen). Recombinant proteins were purified from cell extracts of baculovirus-infected Sf9 cells by using Ni-NTA columns (Qiagen).

### Tyrosine kinase activity assays

Phosphorylation of GST-FLT3S by isolated tyrosine kinases or cell extracts was carried out in a buffer system containing 25 mM Tris–HCl (pH 7.5), 10 mM MgCl_2_, 0.2 mM adenosine 5’-triphosphate, and 2 mM dithiothreitol. Reactions were usually run for 20 minutes at room temperature and were stopped by SDS gel sample buffer. Tyrosine phosphorylation of proteins was determined by immunoblotting analyses with anti-phosphotyrosine antibody PY20, followed by horseradish peroxidase-conjugated secondary antibodies. Detection by the electrochemiluminescence method, capture of immunoblot images, and quantification of band signals were carried out by using FluorChem SP imaging system from Alpha Innotech [14,15]. When the activity assays were performed with GST-FLT3S bound to glutathione-Sepharose beads, reactions were stopped by 15 mM EDTA, and tyrosine phosphorylation was detected by using anti-phosphotyrosine antibody PY20 followed by a fluorescein-conjugated secondary antibody. All microscopic analysis was performed with an Olympus BX51 microscope equipped with a DP71 camera.

## Abbreviations

AML, Acute myeloid leukemia; GST, Glutathione S-transferase.

## Competing interests

The authors declare no conflict of interests.

## Authors' contributions

YC and GY performed the research experiments and wrote the manuscript; JH, WTH performed the research, SL and XF provided research materials and participated in discussion of the research; ZJZ designed the research and wrote the manuscript. All authors read and approved the manuscript.
